# Exploring nurses' perception of taking care of patients with coronavirus disease (COVID‐19): A qualitative study

**DOI:** 10.1002/nop2.616

**Published:** 2020-09-11

**Authors:** Nasrin Galehdar, Tahereh Toulabi, Aziz Kamran, Heshmatolah Heydari

**Affiliations:** ^1^ Social Determinants of Health Research Center Lorestan University of Medical Sciences Khorramabad Iran; ^2^ School of Nursing and Midwifery Lorestan University of Medical Sciences Khorramabad Iran; ^3^ School of Medicine and Allied Medical Sciences Ardabil University of Medical Sciences Ardabil Iran; ^4^ Social Determinants of Health Research Center Lorestan University of Medical Sciences Khorramabad Iran

**Keywords:** COVID‐19, nurses, qualitative study

## Abstract

**Aim:**

Healthcare staff, especially nurses, who are in the frontline of caring of patients with COVID‐19 have valuable experiences about taking care of these patients. Therefore, the aim of this study was exploring nurses' perception of taking care of patients with COVID‐19.

**Design:**

This qualitative study was performed using the conventional content analysis method.

**Methods:**

13 nurses who were taking care of patients with COVID‐19 were selected using purposeful sampling in the Lorestan University of Medical Sciences. The data were collected through telephone interviews and analysed based on the Lundman and Graneheim's approach.

**Results:**

Qualitative analysis of the data revealed 3 sub‐categories, 8 categories and 3 main categories including care erosion, nursing professional growth and necessities.

**Relevance to clinical practice:**

Improve the work situation of the nurses during care of COVID‐19 patients, which can directly or indirectly improve the quality of care of these patients.

## 1 INTRODUCTION

COVID‐19 is an infectious disease caused by a new type of coronavirus associated with the severe acute respiratory syndrome (SARS) (Gorbalenya, 2020). The COVID‐19 was reported as the cause of the outbreak of coronavirus in Wuhan, China (Hui et al., 2020), where it was first reported on 31 December 2019. Due to the rapid outbreak which involved most countries around the globe, the World Health Organization (WHO) declared this disease as a pandemic on 11 March 2020 (WHOa, 2020). The number of people infected with COVID‐19 has reached 4,098,970 with the death toll of 471,519 worldwide until 22 June 2020 (COVID‐19, 2020).

The disease is mainly transmitted through infected micro‐droplets during coughing or sneezing (WHOb, 2020). The incubation period of the disease ranges from 2 to 14 days (Lauer et al., 2020). Constant hand washing, social distancing and following health instructions are the main preventive measures (WHOc, 2020).

Common symptoms include fever, cough and dyspnoea (CDC, 2020). In most cases, the symptoms of the disease are mild as compared to the failure of vital organs such as the lungs, heart and kidneys in some cases (Hui et al., 2020). According to cohort studies, about 80% of patients with COVID‐19 have mild symptoms, half of whom may require hospitalization. About 20% of COVID‐19 patients may experience severe symptoms requiring oxygen therapy or other hospital‐based interventions. Finally, around 5% of patients experience more severe symptoms needing advanced respiratory support by a ventilator in the intensive care unit (ICU) (Wu & McGoogan, 2020). The mortality rate of the disease has been estimated as 1%–5%, but this varies depending on patients' age and health status, especially the presence or absence of underlying diseases (Coronavirus Mortality Rate, 2020).

The first case of COVID‐19 in Iran was observed in Qom on 18 February 2020. Since then, the spread of the disease and its mortality rate have dramatically increased. In Iran, the number of people infected with COVID‐19 has reached 207,522 with the death toll of 9,742 until 22 June 2020 (Coronavirus Pandemic, 2020).

## 2 BACKGROUND

COVID‐19 is a new emerged disease with so many unknown clinical and therapeutic aspects. Although healthcare providers play important roles in confining and controlling the disease and rehabilitating patients, they are at a great risk of being contaminated (Eghbali, Negarandeh, & Froutan, 2020). Among medical staff, nurses are considered as frontline forces fighting against COVID‐19 and are in persistent contact with patients from admission to discharge. Therefore, nurses are exposed to many occupational hazards and psychological pressures as well (Oh et al., 2017). Moreover, the experiences of nurses dealing with the SARS crisis suggest that many problems such as uncertainty, information mismanagement, feelings of anger and guilt, unpreparedness, fear of death, loneliness and physical disorders are common during such catastrophes (Chung, Wong, Suen, & Chung, 2005; Hsu, Chen, Chang, & Chang, 2006; Mok, Chung, Chung, & Wong, 2005).

Lorestan, as one of the most crowded provinces of Iran, is located in the west of the country. In parallel with a national surge in COVID‐19 cases, the incidence of the disease has increased in this province, so that, by July 26, 14,000 cases with COVID‐19 have been confirmed that 350 of them expired. With a population of 1,760,000, Lorestan province has 11 cities, and its capital is Khorramabad. Lorestan University of Medical Sciences, with 18 affiliated hospitals, is responsible for providing health care to patients across the province. During the COVID‐19 pandemic, the Shohadaye Ashayer Hospital, as one of the largest hospitals of the province with 350 active beds, was assigned as the central location for managing the patients. Other counties also devoted parts of their capacities for caring of patients with COVID‐19 disease. According to formal statistics from educational and medical centres, 1981 nurses serve at the forefront of fighting against COVID‐19 in this province (Statistics of nurses, 2020). Given that COVID‐19 is a new emerged disease with many unknown therapeutic, managemental and clinical dimensions, these features need to be divulged, and qualitative researches can be viable choices to scrutinize these fields. The health staff, who are at the frontline of fighting COVID‐19 disease and caring of the patients, can provide researchers with their valuable experiences regarding the disease and its various aspects.

Among healthcare providers, nurses are very precious resources to recognize the disease clinical manifestations, prognostic factors, patients' needs, evidence‐based care practices and nursing management problems during the COVID‐19 crisis.

Nurses have the most contact to the patients with COVID‐19 during hospitalization and spend a lot of time for caring of patients. Explaining nurses' perception of caring of a patient with COVID‐19 may be helpful to improve and promote health of patients. A few studies have been performed on nurses' experiences of caring for patients with COVID‐19. Because of nurses' perceptions are influenced by their caring experiences, and also, considering uncertainties about various aspects of the disease and the fact that the researchers are experts in the qualitative research methodology, and because they are closely involved in taking care of patients with COVID‐19, it is appropriate to use a qualitative research approach. Then, this qualitative study was designed to explore nurses' perceptions towards taking care of patients with this disease. The results of this study can be helpful in facilitating the nurses' work process and caring of patients with COVID‐19.

## 3 METHODS

### 3.1 Design and participants

This qualitative study was conducted with a conventional content analysis approach. The research population was nurses working in the department of inpatient care of COVID‐19 patients in public hospitals affiliated with Lorestan University of Medical Sciences. The inclusion criteria were having at least two weeks of experience in caring of patients with COVID‐19 and willingness to participate in the study. The exclusion criterion was withdrawal from the study for any reason. The participants were selected using the purposeful sampling method based on the years of work experience, the length of experience in the COVID‐19 ward, the wards where the nurses were occupied before COVID‐19 crisis, marital status and the participants' age.

### 3.2 Collecting data

Given the need for immediate data collection to improve patient care process, and the limitations of face‐to‐face contact, data were gathered by 13 semi‐structured in‐depth telephone interviews at a convenient time for the participants during March and April 2020. For the first step, the nurses' information was collected by referring to the nursing offices of respective hospitals. Afterwards, the contacts were made with the selected nurses during their off times.

After explaining the study goals and acquiring verbal consent from the participants, a convenient time for the participant was agreed to conduct the interview. Based on the collected data, the sampling process continued until achieving data saturation in such a way that no new cods from the participants' experiences were emerged.

The duration of the interview was determined based on the participants' desire, patience and experience. All the interviews were recorded by an electronic device. The main question of the study was “Please describe a day of taking care of hospitalized patients with COVID‐19,” or “Please talk about your experiences of taking care of hospitalized patients with COVID‐19.” Afterwards, the interview continued based on the participant's answers with more detailed questions such as "What do you mean?", and "Please explain more about this." The types of questions varied somewhat depending on the participant's position (e.g. caregiver and head nurse) and the type of ward where the nurse was occupied before the crisis. By using probing questions, the researcher guided the interview so as the goals of the research were achieved.

### 3.3 Data analysis

Data analysis was performed simultaneously with interviews based on the proposed methods of Lundman and Graneheim (Graneheim & Lundman, 2004). Immediately after each interview, it was transcribed verbatim. Then, the prepared texts were read several times, and the initial codes were extracted. Related codes were then merged together to form main and sub‐categories according to their similarities and differences. Finally, the concepts hidden in the data were emerged.

### 3.4 Trustworthiness

In order to assure of data trustworthiness, the criteria of credibility, transferability or fittingness, and consistency or dependability were used according to Lincoln and Goba (Ys, 1985). After compiling the initial codes, the participants' opinions were verified for the accuracy of the codes and interpretations, and if the codes contradicted the participants' comments, the codes were corrected accordingly. The control method was performed by two faculty members and experts in the field of qualitative research. After reaching an agreement on the selected codes, they were organized into categories and sub‐categories. Participants also tried to have maximum diversity in terms of knowledge, service life, length of service, service locations during the work period, age and gender.

### 3.5 Ethical considerations

To avoid stress during working time, the interviews were scheduled in a convenient time for the nurses. All the participants were explained about the purpose and manner of the study. Informed consent was obtained from the participants verbally to reduce the risk of disease transmission through potentially contaminated papers and pens. The participants were assured about the anonymity and confidentiality of the information and audio files. They had the right to withdraw during the study at any time. The study was approved by the ethics committee of Lorestan University of Medical Sciences (IR.LUMS.REC.1399.006).

## 4 RESULTS

Eleven females and two males with a mean age and a mean work experience of, respectively, 33 ± 11.4 and 13 ± 8.69 years participated in this study (Table 1). In total, 608 codes were generated which were categorized into 3 sub‐categories, 8 categories and 3 main categories (Table 2). Categories and sub‐categories are explained in what follows.

**Table 1:** Characteristics of participants

Number of Participants	Sex	Level of Education	Ward	Marital Status	Number of Children	Position
1	Female	Postgraduate	Emergency	Single	0	Nurse
2	Female	Bachelor	CCU	Married	1	Head Nurse
3	Female	Student of Postgraduate	CCU	Married	0	Nurse
4	Male	Student of Postgraduate	Emergency	Married	0	Nurse
5	Female	Bachelor	ICU	Married	3	Nurse
6	Male	Student of Postgraduate	Emergency	single	0	Nurse
7	Female	Student of Postgraduate	Infectious	Single	0	Nurse
8	Female	Postgraduate	Emergency	Married	0	Nurse
9	Female	Bachelor	Emergency	Single	0	Nurse
10	Female	Bachelor	Emergency	Single	0	Nurse
11	Female	Bachelor	ICU	Married	1	Nurse
12	Female	Bachelor	ICU		2	Nurse
13	Female	Bachelor	ICU		1	Head Nurse

**Table 2:** Main categories, categories and sub‐categories extracted from the data

Main categories	Categories	Sub‐categories
Care erosion	Bad feeling of inefficiency	
	Dilemma between care delivery and pollution	
	Prisoner in a fence of protective equipment	
	Workload	
Nursing professional growth	The brilliance of the nursing profession	
	The emergence of nursing essence	Love to care Human care
Necessities	The need to support the nurses	
	The need for psychological counselling for the patient	

### 4.1 Care erosion

Data analysis showed that caring of patients with COVID‐19 affected all dimensions of the work and lives of nurses, and this could lead to the gradual disintegration and erosion of the caring of patients over time. In this main category, there were four categories including "bad feeling of inefficiency," "dilemma between care delivery and pollution," " prisoner in a fence of protective equipment" and "workload."

#### 4.1.1 Bad feeling of inefficiency

Data analysis showed that nurses in caring of patients with COVID‐19 experienced a significant level of stress and anxiety. Observing patients' suffering can lead to mental and emotional distress in nurses. The data demonstrated that nurses who witnessed patients passing away would experience psychological pressure. They reported that the death of patients, especially young ones, was unbearable to them. When patients suffer from respiratory distress and the nurse cannot do anything for them, it has many negative effects on the nurse's mood. In this regard, one of the participants reported as following “…It is painful to see a person can't breathe properly in front of your eyes, her heart is being asystole, you know she is suffering, yet you can't do anything…” (10).

#### 4.1.2 Dilemma between care delivery and pollution

Data analysis showed that nurses in caring of patients with COVID‐19, knowing that they are exposed to the infection or that it may transmit the infection to the home environment and cause contamination for themselves and their families. This causes a person to be in a dilemma between choosing of his health and their family or the provision of care to patients with COVID‐19.

Due to the possibility of being carriers, the participants reported that they could not have close contact with their family members, and the fear and anxiety of being a carrier always existed with the nurses when they back home from work. Based on the participants' experiences, some nurses had kids, and they were forced to be separated from their children. One of the participants, who was the mother of a four‐year‐old child, talks about her worries as following “…I'm more worried about my daughter, my daughter is only four years old…children don't have a good understanding of this disease, and you can't tell them much about it… but she always sticks with me. I'm so stressed in a way only God knows; I feel guilty for not letting anyone take she away from me. It's very difficult…” (11).

The participants cited the fear of becoming infected as one of the stressors at workplace. One of the participants in this regard reported “…The first time a patient with COVID‐19 was admitted to our hospital, I had and still have a feeling of intense fear, I was very afraid of the patient himself. In this situation, I have to take care of people who may infect me at any moment…” (10).

#### 4.1.3 Prisoner in a fence of protective equipment

Based on the experiences of the participants, it is difficult and painful for the nurse to wear protective clothes and equipment. The participants reported that wearing the clothes restricted their mobility, increased their body temperature and sweating, and made them feel suffocated. They described the nursing care as difficult while wearing protective clothes. One of the participants noted about the annoyance of protective clothes as following “…We are using our protective equipment now; they are very warm… After ten minutes, you feel like you're in a furnace …” (8). Other participants compared the costumes to astronauts' clothes and mentioned "…We work in these so‐called astronauts' clothes, which is a very difficult and exhausting experience… During the change of clothes, they are completely wet, and we get wet all over, as if we came out of the pool…” (4). The participants warned about the possibility of vision problems, and one of them stated “…It's really difficult to use these glasses, because they aren't medical… and now a lot of colleagues have poor vision…” (4). Another participant said about the annoyance caused by the pressure of the mask on the face “…. The mask we put on our faces during the night shift might last for 12–13 full hours, I even say that after I go home, my nose is bruised…” (5).

Also, according to the participants' experiences, nurses have restrictions on eating, drinking and going to the bathroom during work shifts. When a person wears protective clothing, he or she must keep it until the end of the shift. Due to the limitations of protective clothes, the contaminated environment and the restrictions in going to the bathroom, the nurses may not be able to eat or drink during the shift and even stay NPO until the end of a 12‐hr shift. In addition, due to the environmental contamination and the possibility that nurses may become infected during sleep, many of them do not sleep during 12‐hr night shifts and therefore suffer from sleep disturbances and disordered rest patterns. In this regard, one of the participants described not taking water and food as following “…Yes, we wear protective clothes until the end of the shift, and we don't take them off, and we can't drink water or rest until the end of the shift, and we have to endure until the end of the shift, which is very difficult, especially at 12‐hr night shifts, it's difficult to bear…” (4). She also spoke about the restrictions on going to the bathroom “…It was difficult to go to the bathroom, and we have had limitations” (7).

#### 4.1.4 Workload

Data analysis showed that the nursing care of patients with COVID‐19 was difficult and exhausting. Also, the large volume of services provided and the limitations during the shifts delivered the nurses tired and exhausted. Because patients are unattended, disabled and elders, all the care needs must be met by a nurse. They noted that the multiplicity of patients' care needs and limited number of nursing staff increase the nurses' workload and physical fatigue. One of the participants cited as following “…It really causes fatigue, physical fatigue, our capacity reached three patients, … I was really tired …” (7). Also Data analysis showed that there were many difficulties in managing nursing programmes to provide care for patients with COVID‐19. The participants' experiences during the early days of the spread of COVID‐19 showed they were placed in the workplace according to the same plans as before. They noted that nurses did not have enough skills in managing these patients due to its novel dimensions, and they had to cover many shifts. One of the participants described the pattern of shifting programme as following “…We should have shifted according to the compressed program arranged before Coronavirus outbreak, and in a way, I think it was the weakness of management, and we were annoyed; for example, we used to take care of 22 patients by only 2 nurses, which means 11 patients per nurse, but now the capacity has reached to two or three patients per nurse, and if there were four patients, I really couldn't have figured out anymore…”(7).

The experiences of the participants indicated the occurrence of a wide range of nursing and medical errors during providing services. The participants stated that limited nurses' mobility by wearing protective clothes, heavy workloads, the fear of becoming infected, rushing to take precautions (reducing contact and keeping distance), and stress and anxiety which dominated the work environment might have caused nursing errors. In regard to the high workload and the occurrence of nursing errors, one of the participants stated as following “…By increasing fatigue, the number of errors increased as well…” (7). Another participant commented on maintaining distance with the patient and increasing nursing errors as following “…All of my efforts are to do my job in the shortest possible time to reduce my contact with COVID‐19 patients, so the quality of care will certainly decrease…” (10).

### 4.2 Nursing professional growth

In crisis management, there are always opportunities for individual or professional development, which are considered as positive points of the crisis. Under this main category, there were two categories of "The brilliance of the nursing profession" and "The emergence of nursing essence." These can be considered as opportunities for the development of nursing profession in the COVID‐19 crisis.

#### 4.2.1 The brilliance of the nursing profession

Data analysis showed that upon the spread of COVID‐19 disease, the position of nursing profession has been improved in the view of nurses themselves, officials, patients and communities. The participants reported that although patients with COVID‐19 feel helpless and frustrated, their love and interest towards nurses taking care of them have increased, making them proud of being a nurse. Also, people who are watching the generosity of nurses feel grateful for their efforts in taking care of patients with COVID‐19 and appreciate them in various ways. The participants also noted that caring of patients with COVID‐19 has led to the growth of professional ethics in nursing. One of the participants about the love of the nursing profession stated as following “…I personally feel that I am in a state of war, and that we are serving on the front lines, and this is an honor that has been bestowed on us…” (4). The participants also spoke about positive changes in people's view of the nursing profession as following “…Thank God, during this time, people treat us with great respect and patience, and whoever comes prays for us, and this has encouraged us as we are more focused on the spiritual dimension as well…” (4).

#### 4.2.2 The emergence of nursing essence

Nursing is a profession intercalated with the nature of human beings, and its most important function is to take care of patients and improve their health. Data analysis showed that the COVID‐19 crisis was an opportunity for nurses to become more aware of the depth of their field and profession and to comprehend the true value of nursing in practice. In this category, two sub‐categories of "love to care” and "human care" were identified.

##### Love to care

Data analysis showed that nurses do their job for the sake of God and to improve patients' health. The participants stated that nurses felt very comfortable watching patients recover. They noted that they would not do this because of its financial benefit, and that spiritual feelings and conscience have made them to perform their duties with love and affection in bad working conditions. The participants understand their work as a jihadist action and service in war to defend the people and the homeland. In this regard, one of the participants noted “…That you can help your fellow human beings, you really feel satisfied… You get a spiritual sense…”. The participant goes on to talk about the good sense of supporting patients as following “…When you feel that the patient is calm and trust you, it's a feeling that I will not exchange with the world…” (8). Another participant stated “…but we have had no such a feeling about other patients before…” (5).

##### Human care

Data analysis showed that the nurse is the first person to communicate with the patient, and despite feeling fear and anxiety, she/he gratefully welcomes the patient. Despite all the fatigue and personal problems, nurses take care of patients like a family member, without any expectation. The participants believed that the meaning of real nursing in taking care of patients with COVID‐19 was based on the Nightingale's thought. They described the nurse as a mother who endangers herself only for the sake of her child's health. Addressing the sense of altruism, one of the participants stated "…We had to do suction… we had to put very hot clothes on and go beside patients' beds… it's true that we can't handle the condition sometimes… it's true that they are not of our blood, but they are of our kind…” (11). Another participant noted as following “…We think that our own mother, sister, or child has been admitted to the hospital, and we have the same feeling that we have for our family … (4)”. Another participant described his sense as following “…I hadn't touched the nursing profession so much, and I didn't feel that nursing was that engaging … I recalled the Florence Nightingale's theory stating; I was invited by God…” (5). The same participant stated about human care: “…In my opinion, Watson's theory can be very easily applied to these patients, it is very useful for these patients…” (5).

### 4.3 Necessities

To be successful, a number of factors play a key role and need to be seen in management programmes. The nurse is the most important key force in medical centres and should be given special support. It is also necessary to consider comprehensive care, patient care, and the physical, mental and spiritual dimensions of patients. Accordingly, under the main category of necessities, there were two categories of "the need to support nurses" and "the need to consider psychological counselling for patients."

#### 4.3.1 Necessity of supporting nurses

Analysing the participants' experiences showed that nurses expect comprehensive support from the authorities during these difficult and breathtaking days. The participants noted that officials should support them financially and spiritually. The participants stated that they could barely provide disinfectants and protective equipment outside the workplace. Because of their job and the high possibility of contamination, nurses must disinfect their clothes after their shifts, so they expected authorities to provide disinfectants to them for free. Also, due to the fact that they are portable, nurses need to use protective equipment such as masks in other situations besides working environment as well. In this regard, a participant stated “…For example, one of our relatives, who serve in another office, restated that they have received N95 masks and gloves while we as medical staff who face the danger did not receive any. Why shouldn't we receive such equipment? what is the difference between our family and theirs?” (10).

Regarding the importance of the spiritual support by the authorities, one of the participants noted “…Our staff, and many other doctors and nurses who were not in our center cheered us up and encouraged us a lot…” (8). In regard to the financial support, one of the participants noted “…The colleagues expect to receive the benefits of working amid the Coronavirus outbreak…” (5). Another participant noted about the spiritual support by family members as “… My wife talks to me a lot and gives me hope, saying that you don't have to worry…” (10).

#### 4.3.2 Necessity of psychological counselling for patients

The data content analysis showed that patients experience a lot of pain during their illness. The participants believed that factors such as being away from family members and the fear of death could be very stressful for COVID‐19 patients. So, these patients also need psychological counselling even before the need for nursing care. In this regard, one of the participants stated as following “…When their family is not with them, patients really feel lonely, and all she/he cares about is where my family is, and why doesn't anyone come to visit me? Which sort of disease I have…” (4). Another participant mentioned about patients' concerns as following “…They are somewhat depressed…” (9). Another participant cited patients' fear of death as following “…A 56‐year‐old woman was worried, she told me to tell her family to bring a phone, because she wanted to make a will…” (5). The same nurse reiterated the importance of talking to the patients and mentioned “… I stood next to her for a while, calmed her down, asked her how many children she had and got in touch with her which calmed her down a bit…” (5).

## 5 DISCUSSION

The data quality analysis showed that the nurses experienced many challenges such as bad feeling of inefficiency, stress, excessive physical fatigue, dilemma between care delivery and pollution, and enclosed in protective equipment during taking care of patients with COVID‐19 that all this can lead to decrease of the quality of patient care. However, the outbreak of COVID‐19, along with difficulties and problems for the health staff, especially nurses, has also created opportunities for nurses and the nursing profession to flourish. Improving nurses' occupational status and morale and deepening the understanding of the nursing profession were among the benefits that nursing staff experienced during the outbreak of COVID‐19.

In line with the findings of this study, other studies conducted at the time of the outbreak of SARS were all indicative of psychological challenges and physical fatigue among nursing staff (Chung et al., 2005; Hsu et al., 2006; Mok et al., 2005). Our findings showed that nurses experienced a lot of fear and anxiety when taking care of patients with COVID‐19. In line with these findings, another study showed that healthcare providers experienced a lot of mental disorders such as depression, anxiety, insomnia, and stress that the female nurses and the nurses who were in the frontline of fighting COVID‐19 had more severe psychological problems (Lai et al., 2020). Data analysis showed that the participants' fears and anxieties had different causes. One of these causes was the passing away of a patient with COVID‐19 disease. So, it sounds that nurses can improve taking care of critically ill patients by considering psychological counselling. Another cause of concern and anxiety was the risk of becoming infected. In this regard, the safety infrastructure needs to be strengthened so that nurses deal with no concerns other than patient care.

Other sources of nurses' anxiety were being separated from the family and the fear of possibly infecting family members when they returned home. In this regard, according to reports in China, from 3,000 employees of the health system who had been diagnosed with COVID‐19, at least 22 of them passed away, and some of them also infected their family members (Adams & Walls, 2020; Bai et al., 2020). The healthcare centres' headquarters should take steps to ensure that nurses have the least risk of contamination when they return home. It is also necessary to manage the separation of female nurses from their children with appropriate planning. It sounds that transferring female nurses who have children to wards that are not involved in taking care of patients with COVID‐19 can be effective in resolving this problem.

The results of the data analysis showed that another difficulty for the nurses was physical fatigue due to multiple work shifts. In line with the findings of this study, another study showed that with the increase in the number of patients during the outbreak of COVID‐19, nurses' working hours increased by about 1.5–2 times than the normal. In addition to the increase in working hours, the workload has also increased (Sun et al., 2020). The protective clothes that nurses wear for safety seem to contribute to this great fatigue. According to another study, nurses become tired and exhausted by wearing protective clothes for long periods (Sun et al., 2020). Nurses have to endure heavy and warm clothes until the end of their shifts. It seems that these problems can be resolved by shortening the working hours of nurses, considering rest intervals during shifts and providing them with new sets of protective clothes. By applying this programme, nurses can refresh during her/his rest time by eating, drinking, going to the bathroom and even taking a shower. COVID‐19 may remain a guest of ours for months, and by the number of patients surging every day, nursing programmes should be managed in such a way that nurses can continue taking care of patients upon the coming weeks or months.

According to the data analysis in present study, there have been many inconsistencies in the management of protective equipment and nursing staff during the spread of COVID‐19. This was in line with the findings of another study (Sun et al., 2020). Mismanagement itself can potentially be the source of many other problems. One of these problems plaguing nurses has been inadequate access to the protective equipment. Given that nursing staff are considered as the Achilles' heel of any health organization, if the body of this important cadre is damaged for any reason, the whole system will be damaged, and the health of patients will be endangered. Therefore, necessary arrangements should be made to provide protective clothes for medical personnel, especially nurses. On the other hand, fear and lack of protective equipment can increase the incidence of nursing errors in hospitals. In times of crisis, nursing errors can occur for reasons such as high workload, time constraints, lack of teamwork, individual factors and the complexity of provided service (Zavala, Day, Plummer, & Bamford‐Wade, 2018). Some nursing errors may threaten the patient's life; therefore, the causes of nursing errors in these critical situations must be thoroughly rooted out, and effective measures should be taken to prevent them.

Data analysis showed that in addition to the problems and challenges, there were also opportunities for nurses and the nursing profession during the outbreak of COVID‐19. Improving the professional position and understanding the essence of nursing by nurses were among noteworthy opportunities for nurses during this pandemic. Another study showed that COVID‐19 provided opportunities for professional nursing development, which included such as love, affection, honour, and respect for the profession, appreciation and gratitude towards those around you, and active cooperation during the care of patients with COVID‐19 (Sun et al., 2020). Therefore, it is necessary for the officials and staff of the nursing profession to appreciate this position and redouble their efforts to maintain and improve this position. According to the data quality analysis, nurses need full support from the authorities so that they can keep providing healthcare services during the pandemic. By financial and spiritual supports from the authorities, a part of the efforts of this important stratum of health staff can be compensated. On the other hand, supports from authorities create motivation, love and interest to serve in these critical situations.

It should be considered to apply comprehensive and complete patient care. The data showed that patients were frightened from severe COVID‐19, and many of them accepted death during the course of the disease. In this regard, the experience of the SARS disease also indicates that patients with COVID‐19 may develop degrees of the post‐traumatic stress disorder (Wu, Chan, & Ma, 2005). China's experience in fighting against COVID‐19 also suggests that psychological counselling is essential for patients with this disease (Li et al., 2020). Therefore, it seems that one of the missing links in the care of these patients to be psychological counselling which can be very helpful in managing these critical situations. Therefore, it is recommended that palliative care teams consisting of psychologists and religious experts to be present in all hospitals in order to provide a comprehensive care for COVID‐19 patients (The, 2020).

## 6 LIMITATIONS

One of the limitations of this study was that due to emergency conditions, and the data were collected in the shortest possible time, which limited the time for better validating the data trustworthiness. However, using alternative methods, the validity of the data was provided in some extent. Other limitations of the study included the risk of contamination for the interviewees and the interviewer, and the need to observe social distancing. Because of this, the interviews were conducted by telephone, which could have prevented a deep understanding of the phenomenon. However, researchers have tried to make the interviews as deep and effective as possible.

## 7 CONCLUSION

The study showed that nurses experienced many challenges such as bad feeling of inefficiency, stress, excessive physical fatigue, dilemma between care delivery and pollution and enclosed in protective equipment during taking care of patients with COVID‐19 that all this can lead to decrease of the quality of patient care. One of the sources of nurses' fear and anxiety is the painful death of a patient with COVID‐19. Therefore, it is advisable to consider psychological counselling for nurses so that they can take care of patients in complete calm. Other causes of anxiety in nurses are falling apart from their families and the fear of infecting them in returning home. So, authorities need to consider appropriate precautions to ensure that nurses have the least risk of being contaminated when they go to their homes. Furthermore, patients with COVID‐19 and their families should be provided with psychological comfort by forming palliative care teams consisting of psychologists and religious experts. As the Achilles' heel of any hospital, any problem for nurses can inflict the whole care system and compromise patient care. So, it is necessary for authorities to improve the working place for nurses and augment safety infrastructure so that nurses have no concerns other than patient care. These requirements are fulfilled only through an efficient management approach. As the COVID‐19 disease may stay for months, and given that the number of patients is increasing every day, nursing programmes should be managed in a way to prevent nurses to be exhausted so they can afford continuing work for upcoming weeks or months. The results of this study can pave the path for further researches on how to improve the physical and mental performances of nurses during care of COVID‐19 patients, which can directly or indirectly improve the quality of care of these patients.

## CONFLICT OF INTEREST

The authors declare that they have no competing interests.

## Data Availability

The data sets used during this study are available from the corresponding author on request.
